# Self-management interventions for chronic kidney disease: a systematic review and meta-analysis

**DOI:** 10.1186/s12882-019-1309-y

**Published:** 2019-04-26

**Authors:** Suyuan Peng, Jiawei He, Jiasheng Huang, Longwei Lun, Jiahao Zeng, Shan Zeng, La Zhang, Xusheng Liu, Yifan Wu

**Affiliations:** 1 The Second Medical College of Guangzhou University of Chinese Medicine, Guangzhou, China; 2 Health Science Research, Mayo Clinic, Rochester, MN USA; 3 Renal Division, Peking University First Hospital, Beijing, China; 4 Nephrology Department, Guangdong Provincial Hospital of Chinese Medicine, The Second Affiliated Hospital of Guangzhou University of Chinese Medicine, Guangzhou, China; 5 Chronic Disease Management Department, Guangdong Provincial Hospital of Chinese Medicine, The Second Affiliated Hospital of Guangzhou University of Chinese Medicine, No. 111, Dade Rd, Yuexiu District, Guangzhou, Guangdong Province China; 6 EBM & Clinical Research Service Group, Guangdong Provincial Hospital of Chinese Medicine, The Second Affiliated Hospital of Guangzhou University of Chinese Medicine, Guangzhou, China; 7 Guangdong Provincial Academy of Chinese Medical Sciences, Guangzhou, China

**Keywords:** Chronic kidney disease, Self-management, Chronic disease management

## Abstract

**Background:**

Self-management intervention aims to facilitate an individual’s ability to make lifestyle changes. The effectiveness of this intervention in non-dialysis patients with chronic kidney disease (CKD) is limited. In this study, we applied a systematic review and meta-analysis to investigate whether self-management intervention improves renoprotection for non-dialysis chronic kidney disease.

**Methods:**

We conducted a comprehensive search for randomized controlled trials addressing our objective. We searched for studies up to May 12, 2018. Two reviewers independently evaluated study quality and extracted characteristics and outcomes among patients with CKD within the intervention phase for each trial. Meta-regression and subgroup analyses were conducted to explore heterogeneity.

**Results:**

We identified 19 studies with a total of 2540 CKD patients and a mean follow-up of 13.44 months. Compared with usual care, self-management intervention did not show a significant difference for risk of all-cause mortality (5 studies, 1662 participants; RR 1.13; 95% CI 0.68 to 1.86; I^2^ = 0%), risk of dialysis (5 studies, 1565 participants; RR 1.35; 95% CI 0.84 to 2.19; I^2^ = 0%), or change in eGFR (8 studies, 1315 participants; SMD -0.01; 95% CI -0.23 to 0.21; I^2^ = 64%). Moreover, self-management interventions were associated with a lower 24 h urinary protein excretion (4 studies, 905 participants; MD − 0.12 g/24 h; 95% CI -0.21 to − 0.02; I^2^ = 3%), a lower blood pressure level (SBP: 7 studies, 1201 participants; MD − 5.68 mmHg; 95%CI − 9.68 to − 1.67; I^2^ = 60%; DBP: 7 studies, 1201 participants; MD − 2.64 mmHg, 95% CI -3.78 to − 1.50; I^2^ = 0%), a lower C-reactive Protein (CRP) level (3 studies, 123 participants; SMD -2.8; 95% CI -2.90 to − 2.70; I^2^ = 0%) and a longer distance on the 6-min walk (3 studies, 277 participants; SMD 0.70; 95% CI 0.45 to 0.94; I^2^ = 0%) when compared with the control group.

**Conclusions:**

We observed that self-management intervention was beneficial for urine protein decline, blood pressure level, exercise capacity and CRP level, compared with the standard treatment, during a follow-up of 13.44 months in patients with CKD non-dialysis. However, it did not provide additional benefits for renal outcomes and all-cause mortality.

**Electronic supplementary material:**

The online version of this article (10.1186/s12882-019-1309-y) contains supplementary material, which is available to authorized users.

## Background

Chronic kidney disease (CKD) is a progressive disease that leads to End-Stage Renal Disease (ESRD: maintenance dialysis or kidney transplantation), cardiovascular morbidity and mortality [1]. Approximately 440,000 patients begin dialysis each year worldwide, and annual costs of dialysis and kidney transplants range between US $35,000 and 100,000. This can strain healthcare budgets [2]. Clinical decision making for CKD is challenging [3] due to the heterogeneity of kidney diseases, variability in rates of disease progression, and the competing risk of cardiovascular mortality, the most common cause of death worldwide [4]. Furthermore, CKD is not included in the list of priorities for most non-communicable diseases (NCD), and few countries have clear policies or public programs to prevent and control CKD [5].

CKD management includes slowing the progression to ESRD and decreasing the risk of cardiovascular complications through management of kidney function and CKD progression risk factors such as hypertension and diabetes [6]. In addition to medication, managing risk factors is important clinically because it can prevent, or at least minimize, the likelihood of further renal injury. Long-term CKD management requires a high level of patient involvement, both in decision-making and in the implementation of care. There is growing recognition that patients want to be involved as equal partners in their care. The goal of self-management is to identify strategies that can be used to help patients manage their condition(s) while leading active and productive lives. This includes goal setting, problem solving, symptom management, and shared decision-making, and these strategies are applicable for a diverse population [7]. For patients with CKD, this encompasses a spectrum of behaviors ranging from adherence to medication, exercise, and diet recommendations (self-management maintenance) to recognition of early warning signs, and self-adjustment of home-care regimens.

Despite an established tradition of patient self-management of ESRD, and self-management being a well-established treatment strategy for other chronic conditions such as diabetes [8, 9] and hypertension [10], evidence to support its use for CKD non-dialysis is limited. To lighten the economic burden of ESRD, strategies must be implemented to prevent the progression from early-stage CKD. In this systematic review and meta-analysis, we synthesized results from RCTs to evaluate the effects of self-management intervention on major renal outcomes and mortality in non-dialysis adults with CKD. We also assessed effect modification by proteinuria and blood pressure.

## Methods

We performed a systematic review according to a specified protocol (PROSPERO number: CRD42017059870 [11]). The review reported in accordance with the Preferred Reporting Items for Systematic Reviews and Meta-Analyses (PRISMA) [12] statement recommendations.

### Literature search

Electronic databases were searched using a strategy combining selected MeSH terms with keywords related to CKD and self-management intervention. We used English and Chinese language restriction [11].

Relevant studies were identified by searching the following electronic databases from inception to 12th of May 2018: PubMed, MEDLINE, EMBASE, CINAHL, the Cochrane Library database, the Chinese Biomedicine Database (CBM), Chinese National Knowledge Infrastructure (CNKI), and Wanfang Database. Reference lists from relevant review articles and reviews were also searched.

Studies were first screened according to title and abstract, and the full texts of any study considered relevant according to the selection criteria were assessed for eligibility by 2 independent reviewers (JS. H and JW. H). Disagreements between the reviewers concerning decisions to include or exclude studies were resolved by consensus, and if necessary, consultation with a third reviewer (YF. W).

### Selection criteria

We included randomized controlled trials of self-management intervention compared with usual care for adults (age 18 and above) who had been clinically diagnosed with chronic kidney disease. CKD was defined as a glomerular filtration rate (GFR) < 60 mL/min/1.73 m^2^ or markers of kidney damage, or both, of at least 3 months duration [13]. We included interventions employing self-efficacy training, empowerment, cognitive behavioral therapy, or educational programs focusing on self-management, delivered either face-to-face or through telehealth sessions. Eligible studies had to have been published as full-length articles in peer-reviewed journals. Patients currently receiving renal replacement therapy [RRT] (dialysis or kidney transplantation) were excluded.

### Outcomes

Comparing self-management intervention with the standard CKD treatment during the follow-up period, the primary outcomes of this systematic review included all-cause mortality, number of patients progressing to ESRD, change in GFR, change in proteinuria excretion, and adverse events. Secondary outcomes included health literacy (diet modifications, exercise capacity) and other indexes of CKD risk factors, including glycaemia, blood pressure, blood lipid concentration and C-reactive protein (CRP) level.

### Data extraction and quality assessment

Data extraction included details on the study characteristics (country, study design, sample size and study duration), population characteristics (age, sex, CKD stage), intervention (intervention format, length and delivery) and theoretical frameworks. The comparators and outcomes assessed were extracted, and then tabulated.

We used the recommended Cochrane risk of bias assessment tool [14], and the following items were assessed: random sequence generation, allocation concealment, blinding of participants and personnel, blinding of outcome assessment, incomplete outcome data and selective outcome reporting.

### Data synthesis and analysis

Because of the between-study variance, we used a random-effects model for all analyses [15]. Effects were reported as the relative risk (RR) and 95% confidence intervals (CI) for dichotomous outcomes, and mean difference (MD) or standard mean difference (SMD) and 95% CI for continuous outcomes. The SMD was used when all studies had assess the same outcome, but had measured it differently (e.g. GFR was calculated by CKD-EPI equation or CKD-MDRD equation).

We examined the influence of various characteristics on the study-specific effect estimates by stratifying the analysis by self-management type: a) lifestyle modifications; b) medical-behavior modifications; c) multi-factorial modifications.

Statistical heterogeneity across studies was detected with the Cochrane Q statistic and an *I*^*2*^ test [14]. In cases with substantial heterogeneity, subgroup analysis and meta-regression were conducted to explore potential sources of variation. Subgroup analysis was conducted based on intervention format, treatment duration, and diabetic kidney disease populations.

Funnel plot is a useful tool to visually assess the potential for publication bias. If publication bias had been present, then smaller (less precise) studies that had failed to show statistical significance would have been less likely to have been published. This is reflected as asymmetry in the funnel plot. Publication bias was also examined by visual inspection of funnel plots for asymmetry and either Egger’s linear regression test for dichotomous data [16, 17]^,^ or Harbord’s test [18] for continuous data. A *p*-value less than 0.05 was considered statistically significant. To assess the robustness of our meta-analyses, we conducted a trim and fill analysis. The trim and fill method is used to identify, and correct for, funnel plot asymmetry arising from publication bias.

Data were analyzed using RevMan 5.3.3 and STATA 14.0.

## Results

### Search yield

**Fig. 1:**
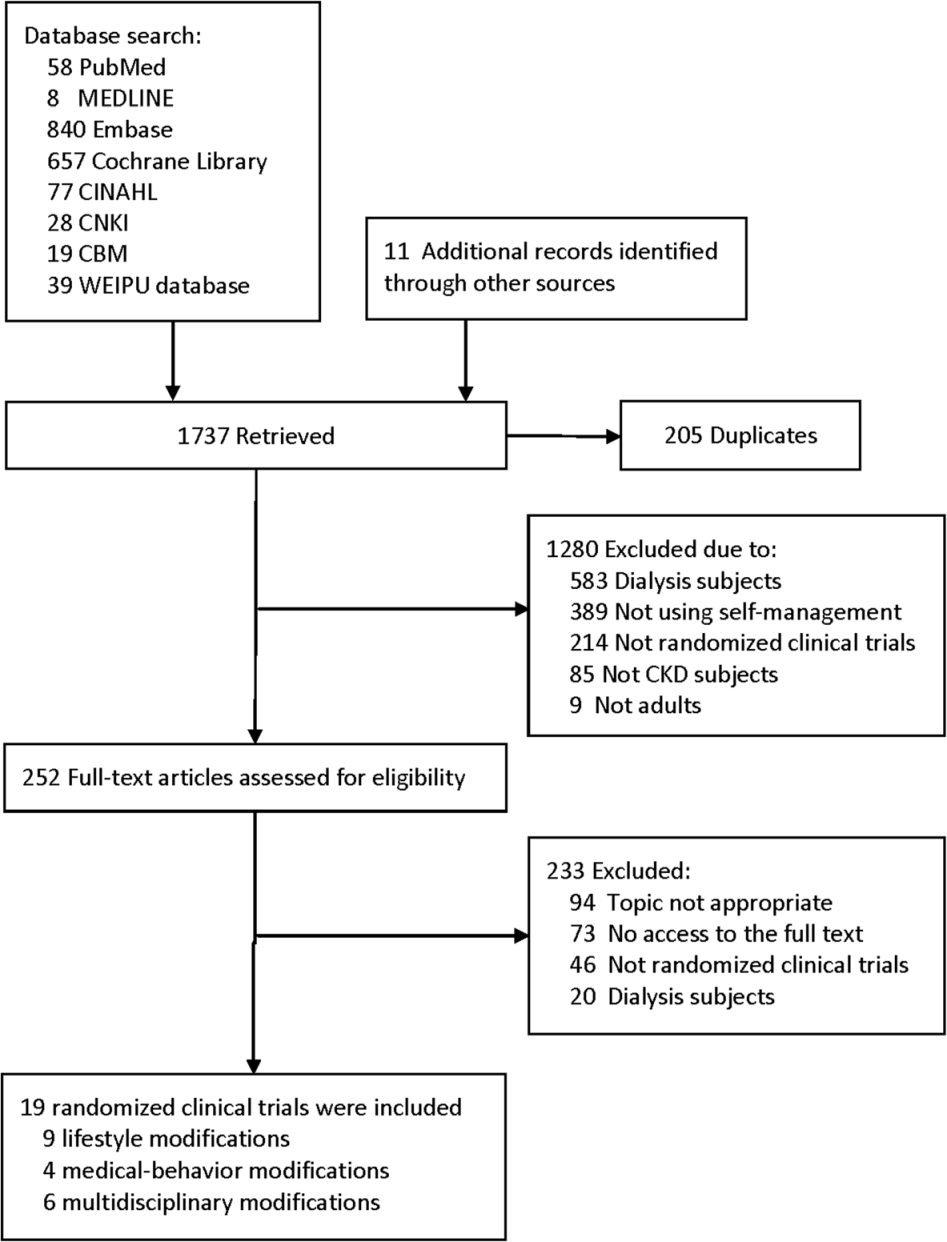
Flow diagram of the stages of article inclusion for this systematic review

Computerized and manual searches resulted in 1737 unique citations, 1280 of which were excluded after reviewing their titles and abstracts. In total, 252 potentially eligible articles were retrieved for full-text review, and 233 articles were excluded. Both reviewers (HS. H. and JW. H.) agreed to include 19 publications in the present study. See Fig. 1 for details on the review process.

### Study and participant characteristics

Nineteen studies, with a total of 2540 CKD patients, were deemed eligible. The clinical and methodological characteristics of each study are summarized in Table 1. The follow-up duration across studies ranged from 3 months to 60 months, and mean duration was 13.44 months. All study participants had CKD, and 10 studies were designed for participants with CKD and concomitant diabetes or hypertension. Studies took place in Europe (2 in the Netherlands, 2 in the UK), North America (3 in the US, 2 in Canada), Asia (2 in Taiwan, 1 in Hong Kong, 1 in Japan), Oceania (4 in Australia, 1 in New Zealand) and Africa (1 in Algeria).

**Table 1:** Characteristics of Studies Included in the Systematic Review

Study	Participants Characteristics	Age	Intervention Format and Delivery	Type of Intervention	Framework	Comparator	Primary Outcomes	Study Duration	Country	Sample size
Meuleman 2017 [47]	CKD1–4(GFR ≥ 20)/Hypertension	55.6 ± 11.7^i^; 54.7 ± 16.0^c^	Sodium restriction; Delivered by health psychologists and dietician	Lifestyle modifications; Face-to-face	Coventry, Aberdeen and London Refined (CALO-RE) taxonomy	Usual care	Sodium intake and BP	38 mo	NED	67^i^, 71^c^
Rossi 2014 [48]	CKD3–4(GFR 15–59)	67.76 ± 12.4^i^; 69.26 ± 12.4^c^	Renal rehabilitation exercise; Delivered by exercise physiologist and physical therapist	Lifestyle modifications; Face-to-face		Usual care	Physical Function Testing, QoL	3 mo	US	59^i^, 48^c^
Teng 2013 [49]	CKD1–3	63.85 ± 12.78	Lifestyle Modification Program; Delivered by clinics’ case managers	lifestyle modifications; Face-to-face	Trans-Theoretical Model (TTM)	Usual care	Diet modification, Exercise	12 mo	Taiwan	52^i^, 51^c^
Mustata 2011 [50]	CKD3–4(GFR15–60)	72.5 (59, 79)^i^; 64 (55, 73)^c^	Exercise; Delivered by physical therapist	Lifestyle modifications; Face-to-face		Usual care	Physical impairment	12 mo	CAN	10^i^, 10^c^
Campbell 2008 [51]	CKD4–5(GFR<30)	69.75 ± 12.15;	Individualized nutritional counseling: providing individualized nutritional counseling (once every 2 weeks), telephone counseling, and self-management principles; Delivered by dietitian	Lifestyle modifications; Telehealth		Usual care	SF-36, SGA	3 mo	AUS	23^i^, 24^c^
Flesher 2011 [52]	CKD3–4(GFR 20–60)/Hypertension	63.4 ± 12.1^i^; 63.4 ± 11.8c	Cooking and exercise programs; Delivered by certified exercise physiologist (CEP), nurse, dietitian, cook educator and exercise physiologist	Lifestyle modifications; Face-to-face	Stanford Patient Education	Usual care	CV risk factors, progression of CKD, self-efficacy & self-management	12 mo	CAN	23^i^, 17^c^
Leehey 2009 [53]	CKD2–4/Diabetes & obesity	66 (range 55–81)	Aerobic exercise	Lifestyle modifications; Face-to-face		Usual care	Proteinuria	6 mo	US	7^i^, 4^c^
Mekki 2010 [54]	CKD2	61 ± 14	Mediterranean diet	Lifestyle modifications; Face-to-face		Usual care	Lipids and apolipoproteins	3 mo	ALG	20^i^, 20^c^
Howden 2015 [55]	CKD3–4(GFR 25–60)/CVD	60.2 ± 9.7^i^; 62.0 ± 8.4^c^	Exercise training and lifestyle program; Delivered by nurse practitioner, exercise physiologist, dietitian, psychologist, credentialed diabetes educator and social worker	Lifestyle modifications; Face-to-face		Usual care	Efficacy, Adherence and Safety	12 mo	AUS	36^i^, 36^c^
Byrne 2011 [56]	CKD1–4(GFR < 90)/Hypertension	62.8 ± 11.8	Evidence-based structured group educational intervention (CHEERS); Delivered by nurse	Medical-behavior modifications; Face-to-face		Usual care	Recruitment, uptake of the intervention and patient satisfaction	6mo	UK	40^i^, 41^c^
van Zuilen 2011 [57]	CKD2–4(GFR 20–70)	58.9 ± 13.1i; 59.3 ± 12.8^c^	Nurse practitioner (NP) care; Delivered by nephrologist	Medical-behavior modifications; Face-to-face		Usual care	Composite nonfatal myocardial infarction, stroke and cardiovascular mortality	60 mo	NED	352^i^, 346^c^
Hotu 2010 [58]	DN(> 0.5 g proteinuria/24 h and Scr 130-300umol/L)& Hypertension	60 ± 7.1^c^; 63 ± 6.6^i^	community visi t(medication adherence and BP control) Delivered by healthcare assistant	Medical-behavior modifications; Face-to-face		Usual care	Change in BP.	4.5 mo	NZ	30^i^, 28^c^
Williams 2012^b^ [59]	CKD2–4/T1/T2DM&CVD	74.31 ± 8.37	multifactorial intervention designed to improve medication self-efficacy and adherence; Delivered by nurse	Medical-behavior modifications; Face-to-face & Telehealth	Health Belief Model (HBM)	Usual care	Medication self-efficacy & adherence	12 mo	AUS	24^i^, 24^c^
Joboshi 2017 [60]	CKD1–5	67 ± 11.5^i^; 70.1 ± 11.1^c^	Participants’ behavioral targets included blood pressure management, medication management, and nutritional management of salt and potassium intakes; Delivered by nurse	Multifactorial modifications; Face-to-face		Usual care	Self-efficacy and self-management behavior	3 mo	JPN	32^i^, 29^c^
Ishani 2016 [61]	CKD3–5(GFR < 60)	75.1 ± 8.1	Telehealth and interprofessional case management (BP, volume status, proteinuria, diabetes mellitus, lipid levels, and depression; health literacy and patient activation); Delivered by nephrologist, nurse practitioner, nurses, clinical pharmacy specialist, psychologist, social worker, telehealth care technician and dietician	Multifactorial modifications; Telehealth	Components of the chronic care model(CCM).	Usual care	Death, hospitalization, emergency department visits, or admission to skilled nursing facilities	4.5 mo	US	450^i^, 150^c^
Steed 2005 [62]	CKD1–5/T2DM and microalbuminuria	59.2 ± 8.8^i^; 60.3 ± 8.6^c^	Diabetes self-management and developing problem solving techniques (self-monitoring of blood glucose, diet, exercise and medication) Delivered by diabetes nurse, dietician	Multifactorial modifications; Face-to-face		Usual care	QoL	3 mo	UK	59^i^, 65^c^
Williams 2012^a^ [63]	CKD3–5/Diabetes	68 ± 8.3^i^; 66 ± 10.8^c^	BP & medication adherence; Delivered by renal specialist nurse	Multifactorial modifications; Face-to-face & Telehealth	Health Belief Model (HBM)	Usual care	BP control, medication adherence	12 mo	AUS	36^i^, 39^c^
Chan 2009 [64]	Scr 150-350umol/l /T2DM	65 ± 7.2	Treatment compliance and self-care (drug use, insulin injection, self-monitoring of blood glucose, and lifestyle modification); Delivered by dietitian and doctor-nurse team	Multifactorial modifications; Face-to-face		Usual care	Death and/or renal end point (Cr > 500umol/L)	24 mo	HK	81^i^, 82^c^
Chen 2011 [65]	CKD3–5	68.39 ± 12.08	Interactive individualized education sessions; Delivered by CKD nursing specialists	Multifactorial modifications; Face-to-face & Telehealth	SMS program	Usual care	Improved GFR, No. ofhospitalizations	12 mo	Taiwan	27^i^, 27^c^

*AUS* Australia, *US* United States, *GCG* Greater China Group (Mainland China, Hong Kong, Macau and Taiwan), *CAN* Canada, *NED* Netherlands, *UK* United Kingdom, *ALG* Algeria, *NZ* New Zealand, *JPN* Japan

*T1DM* Type 1 Diabetes, *T2DM* Type 2 Diabetes, *Mo* Months

Lifestyle modification, targeting nutrition management, weight management or physical exercise; Medical-behavior modification, targeting medicine adherence, disease cognition and complication control; Multi-factorial modifications, combine lifestyle and medical behavior;

^I^intervention; ^c^Control group

### Intervention features

Trials in this review comprised various kinds of self-management. We grouped trials into similar interventions (lifestyle modifications; medical-behavior modifications and multi-factorial modifications). Lifestyle interventions were the most common, followed by medical related practice and multi-factorial interventions. Among the lifestyle intervention trials, 9 studies included interventions related to lifestyle modification, targeting nutrition management, weight management or physical exercise. Four studies included in interventions were related to medical-behavior modification, targeting medicine adherence, disease cognition and complication control; and 6 were related to multi-factorial modifications (combined lifestyle and medical behaviors). The programs were delivered either face-to-face in an individual or group format, or via telehealth sessions (i.e. telephone, Digital Versatile Disc). They were conducted by a range of professionals, including nurses, dieticians/nutritionists, certified exercise physiologists (CEP) and physicians (Table 1).

Six different self-management intervention theoretical frameworks were included in these articles: the Coventry, Aberdeen, and London—Refined (CALO-RE) taxonomy; components of the chronic care (CCM) model; the Trans-Theoretical Model (TTM), the Stanford Patient Education, the SMS program, and the Health Belief Model (HBM). When there was insufficient information, we attempted to contact the authors, but the response rate was poor.

### Quality assessment

The risk of bias of included studies is summarized in Additional file 1: Figure S1 and S2. The main cause of potential bias was inadequate allocation concealment. Due to insufficient information, judgement could not be made in most of the studies regarding either allocation concealment or selection reporting.

### Effects of self-management intervention on kidney disease progression

**Fig. 2:**
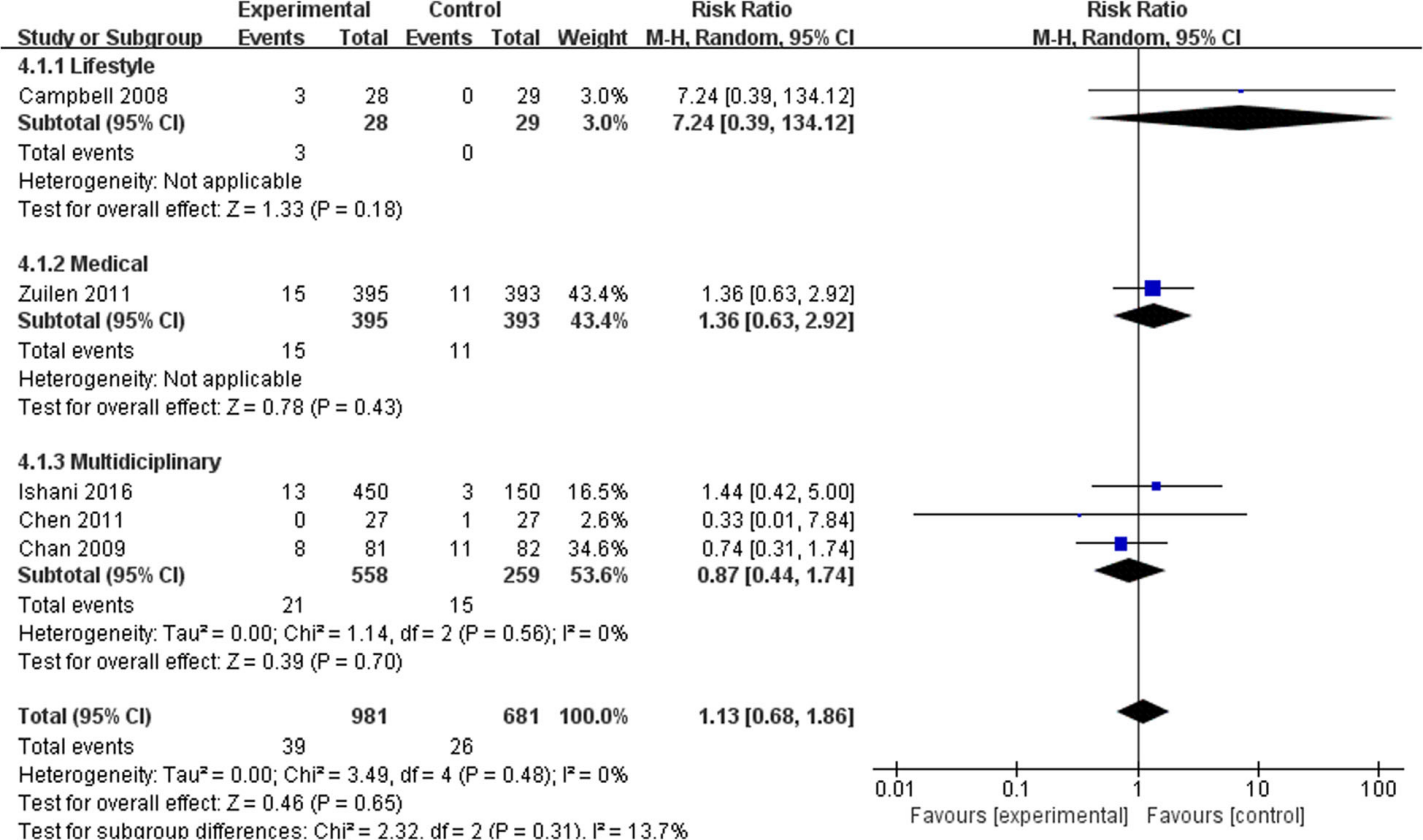
Pooled Estimates Comparing Self-management Intervention with Usual Care for All-cause Mortality; M-H, Mantel-Haenszel method; IV, independent variable method

**Fig. 3:**
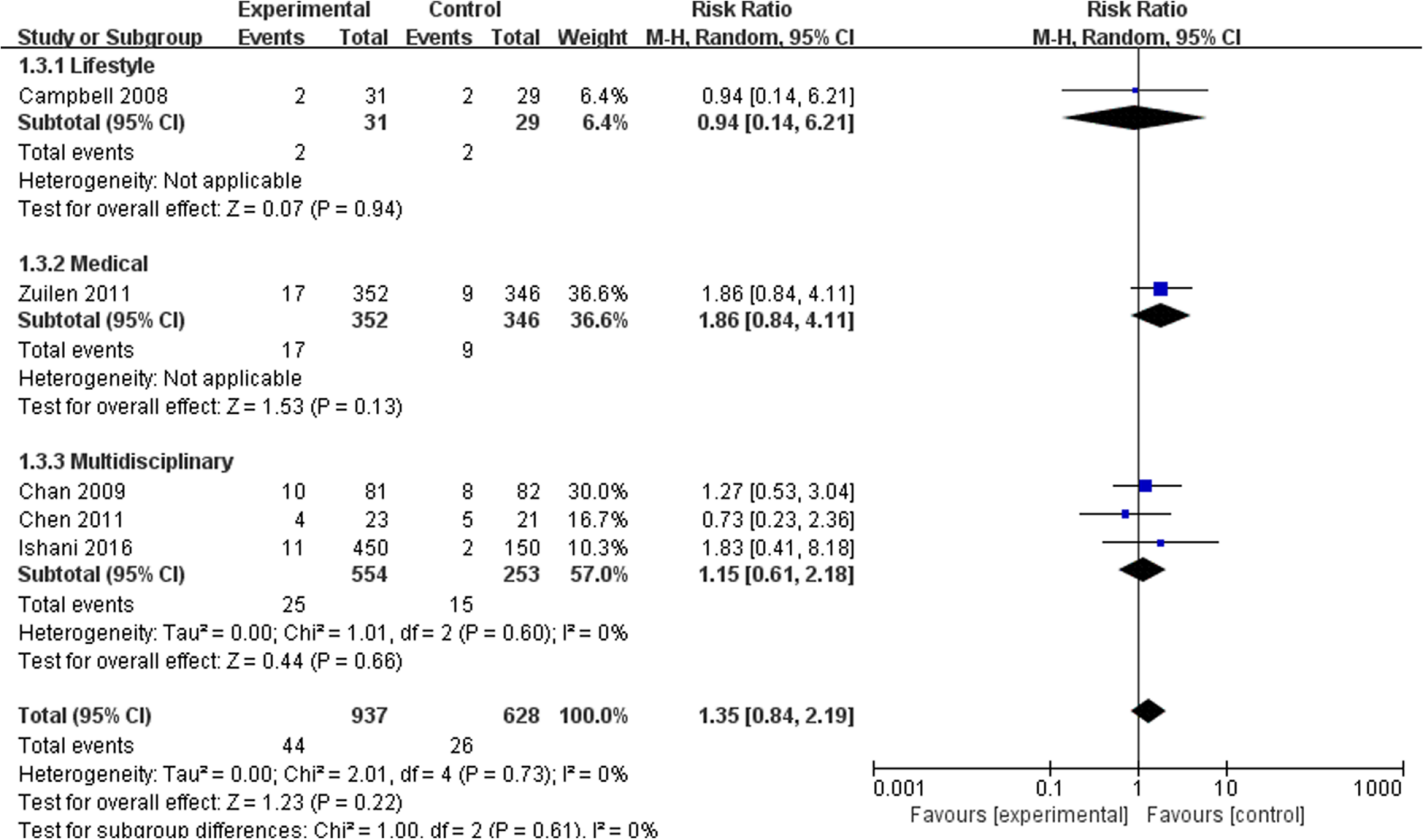
Pooled Estimates Comparing Self-management Intervention with Usual Care for Risk of Dialysis; M-H, Mantel-Haenszel method; IV, independent variable method

**Fig. 4:**
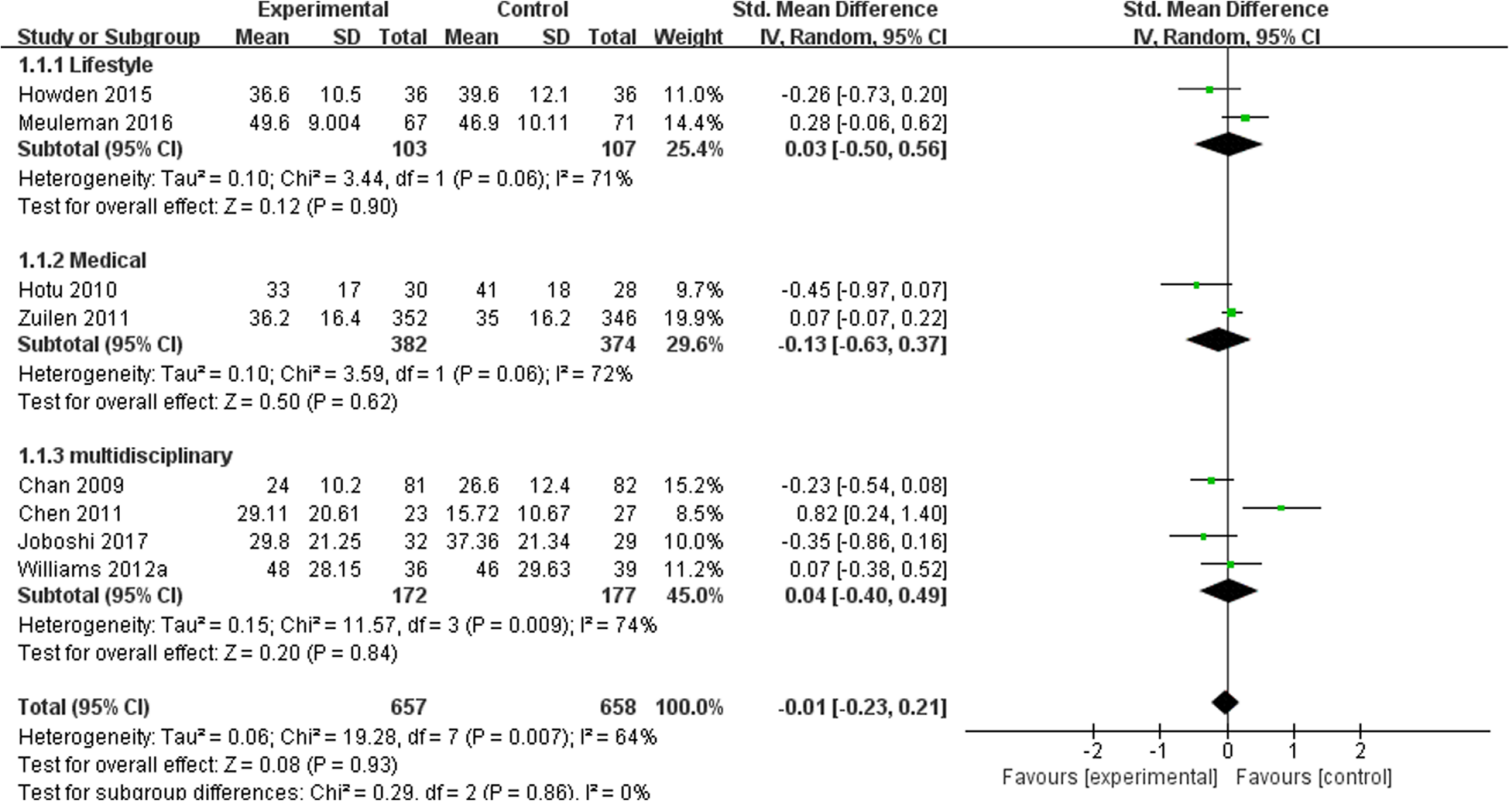
Pooled Estimates Comparing Self-management Intervention with Usual Care for Changing on GFR; M-H, Mantel-Haenszel method; IV, independent variable method

**Fig. 5:**
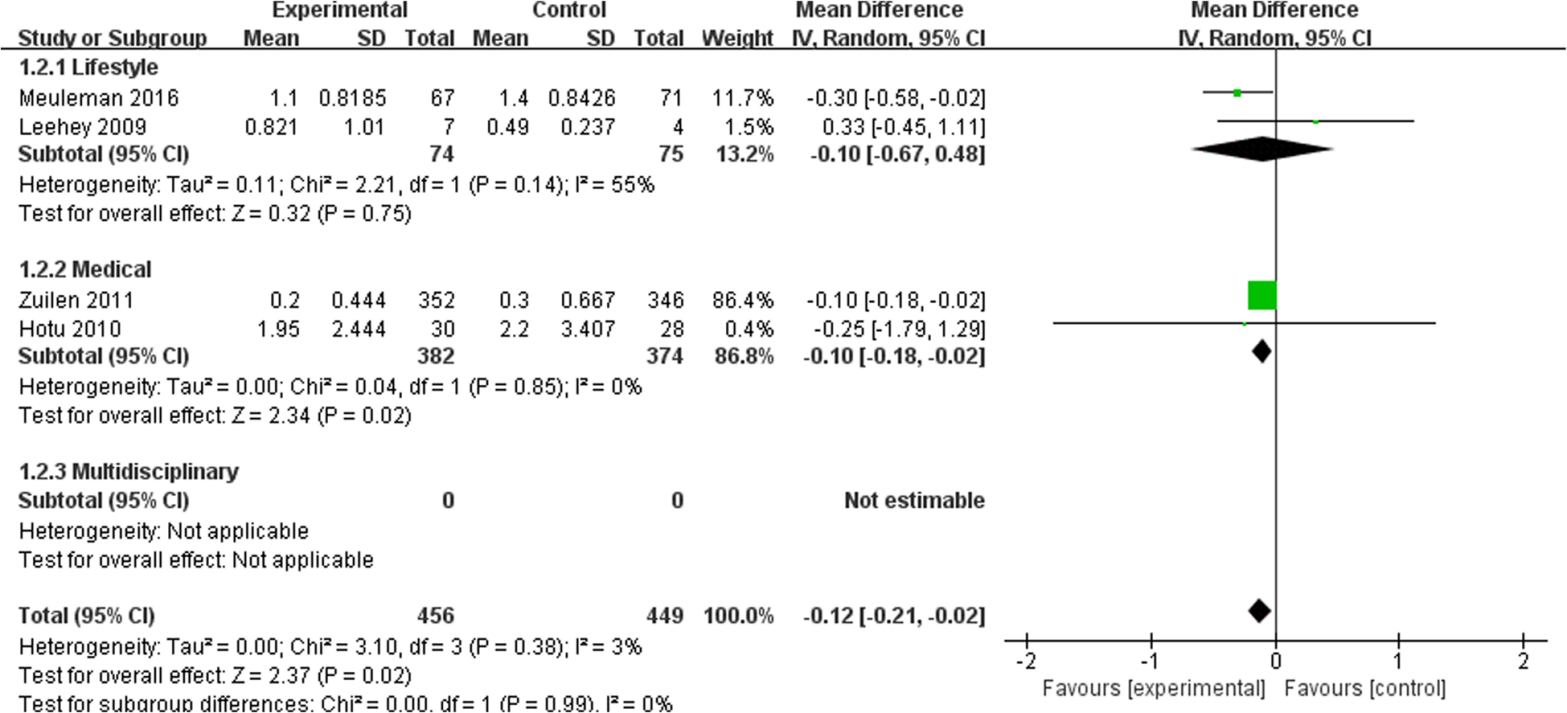
Pooled Estimates Comparing Self-management Intervention with Usual Care for 24 h Urinary Protein Excretion; M-H, Mantel-Haenszel method; IV, independent variable method

Figures 2, 3, 4 and 5 shows the pooled estimates for the primary outcomes. Compared with the standard treatment strategy, self-management intervention showed no significant difference in risk of all-cause mortality (5 studies, 1662 participants; RR 1.13; 95% CI 0.68 to 1.86, I^2^ = 0%) (Fig. 2), risk of dialysis (5 studies, 1565 participants; RR 1.35; 95% CI 0.84 to 2.19, I^2^ = 0%) (Fig. 3), or change in GFR (7 studies, 1315 participants; SMD -0.01; 95% CI -0.23 to 0.21, I^2^ = 64%) (Fig. 4). Moreover, self-management interventions were associated with **a lower 24 h urinary protein excretion** than that of the usual care (4 studies, 905 participants; MD − 0.12 g/24 h; 95% CI -0.21 to − 0.02, I^2^ = 3%) (Fig. 5).

The funnel plots, Egger’s regression asymmetry test, and Harbord’s regression asymmetry test each indicated no significant publication bias for any outcome. There was also no statistical heterogeneity for any of the outcomes (Additional file 1: Figure S3).

We also conducted a trim and fill analysis to determine the robustness of our meta-analysis. There was no effect of replacing missing studies, and the results showed that these estimates were robust and changed little (Additional file 1: Table S1).

### Effects of self-management on risk factors

For the surrogate outcomes, meta-analysis showed that self-management interventions were associated with **a lower blood pressure level** (SBP: 7 studies, 1201 participants; MD − 5.68 mmHg; 95% CI -9.68 to − 1.67; I^2^ = 60%; DBP: 7 studies, 1201 participants; MD − 2.64 mmHg, 95% CI -3.78 to − 1.50; I^2^ = 0%), and **a lower CRP level** (3 studies, 123 participants; SMD -2.8; 95% CI -2.90 to − 2.70, I^2^ = 0%) than that of the usual care. However, there were no statistical differences in HbA1c or total cholesterol (TC) levels. Likewise, for the behavioral risk factor outcomes, patients who had received exercise management had **longer distances on the 6-min walk** (3 studies, 277 participants; SMD 0.70; 95% CI 0.45 to 0.94; I^2^ = 0%) than the control group. Regarding body weight and BMI, diet management appeared no better than usual care alone.

In the stratified analysis, it was observed that the **multi-factorial modification group** was associated with a significant **decrease in HbA1c** (3 studies, 344 participants; MD -0.68%; 95% CI -0.99,-0.36; I^2^ = 65%) compared with the usual care. (Additional file 1: Table S2).

### Adverse events of self-management intervention

Four studies recorded adverse events (AEs); there were no specific descriptions for definition of all AEs; and no AEs occurred during the follow-up periods.

### Meta-regression and subgroup analyses

Table 2 lists the results of univariate meta-regression analyses for exploring the potential sources of between-study heterogeneity. Though this review did not find evidence of specific contributors to heterogeneity, these effects might have differed according to baseline CKD stage, race, or the prevalence of disease.

**Table 2:** Univariate Meta-regression for Effects of Self-management Intervention on Primary Outcomes

	All-cause mortality				Risk of dialysis				Change in GFR				24 h urine protein			
Covariates	No.	R^2^, %^a^	*P* Value^b^	β (95% CI)	No.	R^2^, %	*P* Value	β (95% CI)	No.	R^2^, %	*P* Value	β (95% CI)	No.	R^2^, %	*P* Value	β (95% CI)
Age																
≥65y	4	0.00	0.56	0.34[−1.37,1.99]	4	0.00	0.38	0.52[− 1.10,2.14]	4	0.00	0.77	−0.09[−0.82,0.63]	3	0.00	0.37	0.49[−1.32,2.29]
<65y	1				1				4				1			
Treatment duration																
>12 m	3	0.00	0.74	−0.41[−4.09,3.26]	3	0.00	0.276	0.78[−1.08,2.63]	3	0.00	0.76	0.09[−0.63,0.82]	2	0.00	−0.96	−0.36[−2.0,1.25]
≤12 m	2				2				5				2			
Diabetic kidney disease																
CKD	4	0.00	0.32	−0.64[− 2.37,1.08]	4	0.00	0.902	−0.07[−1.82,1.67]	5	9.11	0.32	−0.30[− 0.97,0.38]	2	0.00	0.44	0.36[−1.25,1.97]
DKD	1				1				3				2			

^a^R^2^ indicated the proportion of between-study variance explained by the model. ^b^*P* value represented to *P* value of Q model. *P*<0.05 indicated a between-group difference of the effects of self-management intervention for the covariate

## Discussion

Although RRT has been available for decades in wealthy countries, most people with kidney failure have insufficient access to life-saving dialysis and kidney transplants. CKD care is an effective alternative, yet it has limitations: underutilization of health professionals in the care of patients with CKD, limited capacity for CKD surveillance, a general absence of national strategies to support CKD care, and poor integration of CKD care programs into national NCD control schemes [19]. A global change in the approach to CKD, from treatment to prevention, is imperative, especially in low- and middle-income countries that lack resources for RRT^2^.

### Results in relation to other studies and reviews

To date, 6 systematic reviews have studied CKD management in general. While Lopez-Vargas 2016 [20], and Galbraith 2018 [21] provided education-based interventions, they did not focus on changing participants’ beliefs. Although interventions might have been necessary for education, they are often insufficient, on their own, to produce behavioral change [22]. LEE (2016) [23] and Lin (2017) [24] identified CKD as including ESRD patients. In their study, dialysis participants’ self-management intervention showed significant effects on self-efficacy, depression and health-related quality of life, while the effectiveness of self-management of non-dialysis CKD patients is limited. Helou (2016) [25] focused on multi-factorial management of diabetic kidney disease (DKD) patients. A recent systematic review has identified only 5 studies, and they are of varying methodological quality. This has led the authors to conclude “the effect of self-management programs in CKD (Stages 1–4) cannot be conclusively ascertained [26].”

Cardiovascular disease events, proteinuria and diabetes are associated with CKD progression, and the former have been the major cause of death in those with CKD. In our study, the results showed a significant drop in blood pressure and a lower 24 h urinary protein excretion among the self-management group. Long-term blood pressure drops reduce proteinuria and other indicators of structural damage. Early change in proteinuria may lead to an increased risk of ESRD and early death, and may also be associated with slower progression of kidney disease [27]. In the stratified analysis, it was observed that the multi-factorial modification group was associated with a significant decrease in HbA1c. Compared with CKD patients, co-existing CKD and diabetes patients carry a poor prognosis with increased all-cause mortality and cardiovascular mortality [28].

We also found that self-management intervention led to additional kidney protection, due to lower CRP levels and better exercise capacity. Evidence shows that exercise training results in improved physical performance and functioning among patients with CKD [29]. Also, regular participation in moderate-intensity exercise may enhance certain aspects of immune function and exert anti-inflammatory effects [30, 31]. Inflammatory markers such as CRP and anti-inflammatory cytokines correlate with underlying causes and consequences of the inflamed uremic phenotype such as oxidative stress, endothelial dysfunction, CVD, infections and protein-energy malnutrition (PEM, also referred to as PEW) [32]. As inflammatory biomarkers are sensitive predictors of outcomes in patients with ESRD, inflammation appears to be a target for preventive and therapeutic interventions in patients with CKD [33, 34]. This is consistent with our findings.

### Self-management theoretical frameworks

Patient-oriented self-management is the cornerstone of chronic disease management, and optimized self-management is fundamental to controlling risk factors and improving disease management. Seeking to facilitate behavioral change rather than providing a purely educational program [35], self-management requires patients to shift away from passive education, and to become responsible for their own illness [36]. Patients are no longer a passive recipient of education; they are an active determiner of their health. Self-management intervention is a vehicle for helping patients develop skills and techniques to enhance self-care of their chronic conditions. Changing patients’ beliefs is usually measured by asking “how confident are you” or “how sure are you” that under specific conditions they can achieve certain behaviors or physiological states.

Our study consisted of the following 6 theoretical frameworks, with each model having unique aspects. These ranged from uni-dimensional variables to complex multi-dimensional constructs. Noar [37] analyzed the components of health behavior frameworks in terms of structures appertaining to attitudinal beliefs; self-efficacy and behavioral control beliefs; normative beliefs; risk-related beliefs and emotional responses; and intention, commitment and planning. We have updated the table after Noar (see Additional file 1: Table S3) showing how the structure and content of the models can be understood on multiple levels. The aforementioned theoretical frameworks for chronic diseases help refine the theoretical basis of intervention evaluations. However, at present, none are commonly used outside of research settings. Furthermore, the effectiveness of self-management for early-stage CKD is limited, and requires additional large-sample RCTs to assess the effectiveness of self-management intervention [38].

Additionally, computer-based machine learning algorithms can identify intervention at a practice level in real time to allow more focused and immediate correction of bias in NCD management [39]. For example, several studies have adopted novel machine learning algorithms to perform knowledge discovery on management of AEs or cancer complications [40, 41]. Therefore, the machine learning approach provides a general way to discover self-management strategies for NCDs [42].

### Strengths of this study

This study has several strengths. First, the concept of self-management is debatable. Many prior studies have failed to distinguish it from health education or chronic disease management. Additionally, there are a variety of risk factors for CKD. Our study grouped similar risk factors together, and found that the effect of comprehensive intervention (lifestyle combined with medical behavior) is more effective. We believe this will be a new trend in future self-management intervention.

Second, current reporting of intervention content in published research articles and protocols lacks consistent terminology, making replicability difficult and uncommon. We concluded that there are 6 types of self-management frameworks, and this can provide reference for future self-management decision making.

### Limitations

Our study also has several limitations. First, the effects of interventions on lifestyle and risk factor modification may require years for their results to modify surrogate and hard outcomes. A methodological limitation of the studies was the short-term follow-up might not distinguish kidney outcome differences, with most of the included studies had a follow-up time shorter than 2 years. Second, there was heterogeneity in patient characteristics, trial designs and risk factor targets (obesity, hypertensive, salt intake, etc.) among the included studies. The number of included studies also limited the power for further exploration with multi-variate meta-regression or multi-level subgroup comparisons [43]. Therefore, we could only partially explain the influences of blood glucose on intervention effects. Third, funnel plots and Egger’s test did not suggest publication bias, owing to the included studies (published studies only); such bias could still exist. Fourth, for the self-management framework, there is extensive heterogeneity in the body of research available, and it is uncertain what theory is best to predict (and ultimately to change) health behavior. Therefore, more integrative approaches are needed. Finally, only Chinese and English language reports were included. Consequently, we may be missing data from important studies published in other languages.

The drawback of a manual literature review is the time-consuming step of screening articles to select those that fulfill the requirements. Accordingly, some studies have leveraged computer-based topic analysis approaches to support literature review [44, 45]. In the future, these approaches could be leveraged to facilitate the efficiency and effectiveness of systematic review [46].

## Conclusion

We observed that self-management intervention provided additional benefits for neither renal outcomes nor all-cause mortality, when compared with standard treatments during a follow-up of 13.44 months in patients with CKD non-dialysis. However, it does show benefits for urine protein decline, blood pressure level, exercise capacity and CRP level. Hence, self-management intervention was beneficial for changing modifiable risk factors (e.g. proteinuria, blood pressure level, blood glucose level, exercise capacity) for the progression of kidney disease. It may have been beneficial in optimizing CKD patient outcomes and avoiding progression to ESRD, and thus may have improved survival.

Integration and ensuring the sustainability of healthcare self-management plans requires a large sample of RCT research and a unified and precise self-management intervention framework. These resources will help determine the ideal implementation for interventions.

## Additional file

Additional file 1: **Figure S1.** Summary for Risk of Bias of Included Studies. **Figure S2.** Risk of Bias Graph of Included Studies. **Figure S3.** Funnel Plots, Contour-enhanced Funnel Plots, and Egger/Harbord Regression. **Table S1.** Association between Self-management Intervention and Standard Care by Subgroups. **Table S2.** Effects of Self-management on CKD Risk Factors. **Table S3.** Structure and Content of Self-management. (DOC 268 kb)

## Abbreviations

AEs: Adverts events
CEP: Certified exercise physiologists
CI: Confidence interval
CKD: Chronic kidney disease
CRP: C-reactive Protein
DKD: Diabetic kidney disease
eGFR: Estimated Glomerular Filtration Rate
ESRD: End-Stage Renal Disease
KDIGO: Kidney Disease: Improving Global Outcomes
MD: Mean difference
MeSH: Medical Subject Headings
PEW: Protein-energy malnutrition
PRISMA: Preferred Reporting Items for Systematic Reviews and Meta-Analyses
RCT: Randomized controlled trial
RR: Relative risk
RRT: Renal replacement therapy
SMD: Standard mean difference
T1DM: Type 1 Diabetes
T2DM: Type 2 Diabetes
TC: Total cholesterol
