# Supplementary material for: Self-management interventions for chronic kidney disease: a systematic review and meta-analysis
Source: BMC Nephrol. 2019 Apr 26;20:142. doi: 10.1186/s12882-019-1309-y (PMC6486699; doi:10.1186/s12882-019-1309-y)
Supplement: Supplementary file 1 — Figure S1. Summary for Risk of Bias of Included Studies. Figure S2. Risk of Bias Graph of Included Studies. Figure S3. Funnel Plots, Contour-enhanced Funnel Plots, and Egger/Harbord Regression. Table S1. Association between Self-management Intervention and Standard Care by Subgroups. Table S2. Effects of Self-management on CKD Risk Factors. Table S3. Structure and Content of Self-management. (DOC 268 kb) [file 12882_2019_1309_MOESM1_ESM.doc]

Additional file 1: Figure S1. Summary for Risk of Bias of Included Studies.
The green symbols represent low risk of bias, the yellow symbols represent unclear risk of bias, and the red symbols represent high risk of bias. The figure was generated using Review Manager Version 5.3.3.


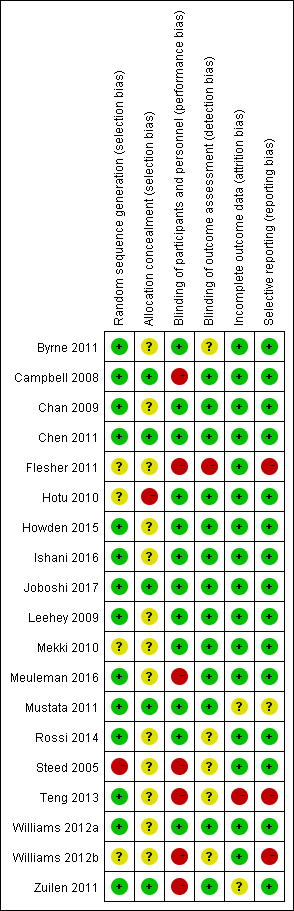


Additional file 1: **Figure S2. Risk of Bias Graph of Included Studies.**
Each methodological quality item is presented as percentages across all included studies. The figure was generated using Review Manager Version 5.3.3.


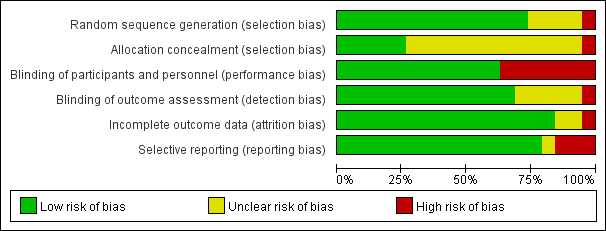


Additional file 1: **Figure S3.** **Funnel Plots, Contour-enhanced Funnel Plots, and** **Egger/Harbord Regression
Asymmetry Test for Assessment of Publication Bias.**(A) All-cause mortality, (B) Risk of dialysis, (C) Change in GFR, (D) D.24h Urine Protein, for comparisons between self-management intervention and standard care. For contour-enhancd funnel plots, contours in black are regions of studies with P values between 0.1–0.05, those in dark gray are P values between 0.05-0.01, and those in gray are
P values < 0.01. If studies appear to be missing in areas of high statistical significance, then publication bias is a less likely cause of the funnel asymmetry, and vice versa.

| 1. **All-cause mortality** |  |
| --- | --- |
| **Contour-enhancd funnel plots** | **Harbord’s Regression Asymmetry Test (*P*=0.96)** |
| **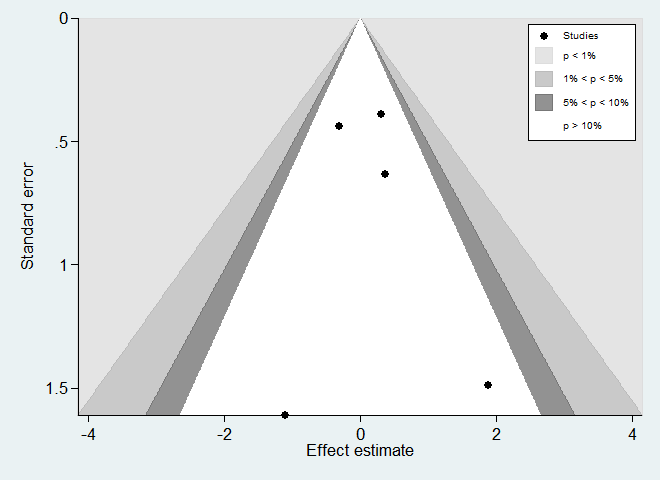** | **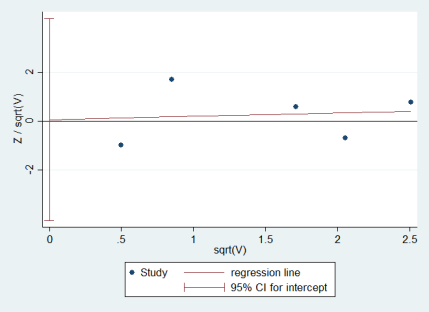** |
| 1. **Risk of dialysis** |  |
| **Contour-enhancd funnel plots** | **Harbord’s Regression Asymmetry Test (*P*=0.18)** |
| **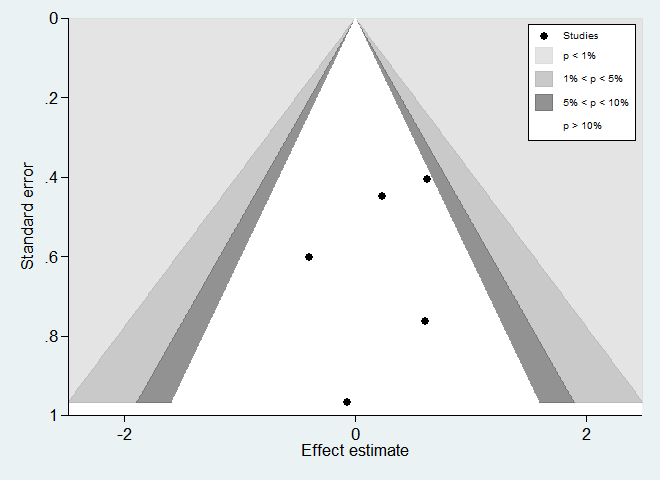** | **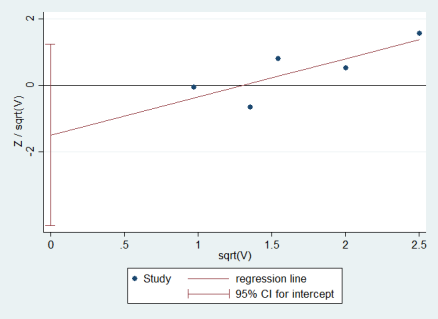** |
| 1. **Change in GFR** |  |
| **Contour-enhancd funnel plots** | **Egger’s Regression Asymmetry Test (*P*=0.70)** |
| **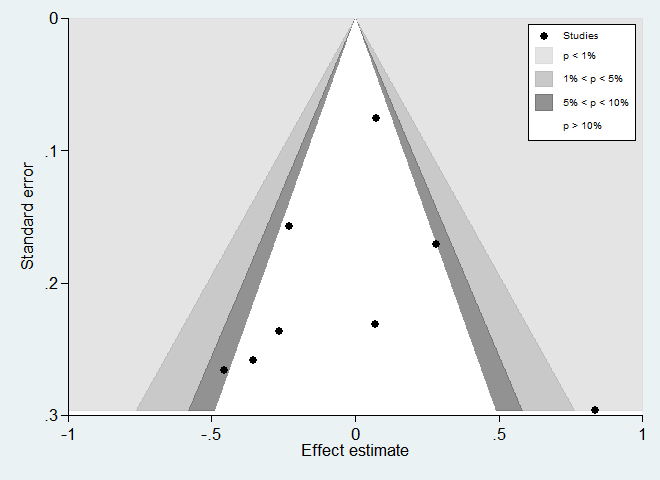** | **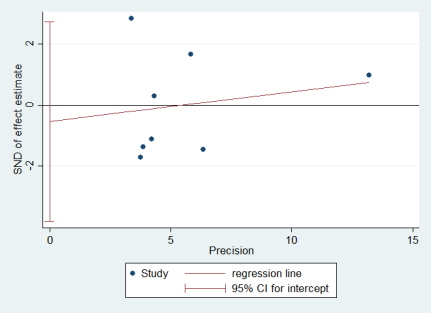** |
| 1. **24h Urine Protein** |  |
| **Contour-enhancd funnel plots** | **Egger’s Regression Asymmetry Test (*P*=0.66)** |
| **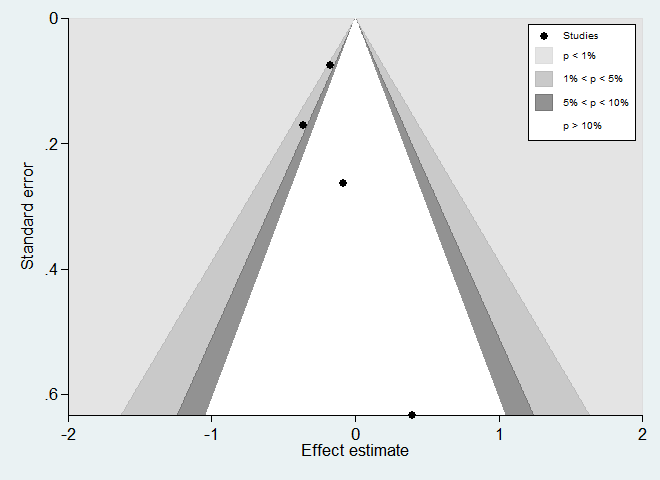** | **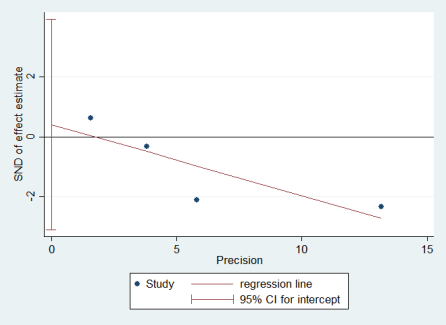** |

Additional file 1: **Table S1 Association between Self-management Intervention and Standard Care by Subgroups**

|  | **All-cause mortality** | |  | **Risk of dialysis** | |  | **Change in GFR** | |  | **24h urine protein** | |  |
| --- | --- | --- | --- | --- | --- | --- | --- | --- | --- | --- | --- | --- |
|  | **No.** | **Random effects Model RR (95% CI)** | **Heterogeneity statistics p-value, I^2^%** | **No.** | **Random effects Model RR (95% CI)** | **Heterogeneity statistics  p-value, I^2^%** | **No.** | **Random effects  Model SMD (95% CI)** | **Heterogeneity statistics  p-value, I^2^%** | **No.** | **Random effects Model MD (95% CI)** **(g/24h)** | **Heterogeneity statistics  p-value, I^2^%** |
| **Overall summary** | 5 | 1.13[0.68,1.86] | 0.51,0 | 5 | 1.35[0.84,2.19] | 0.73,0 | 8 | -0.01[-0.23,0.21] | 0.01,64 | 4 | -0.12 [-0.21,-0.02] | 0.68,3 |
| **Age** |  |  |  |  |  |  |  |  |  |  |  |  |
| ≥65y | 4 | 0.9[0.50,1.90] | 0.40,0 | 4 | 0.9[0.53,1.63] | 0.74,0 | 4 | -0.02[-0.29,0.24] | 0.06,59 | 3 | 0.117[-0.20,-0.04] | 0.40,0 |
| ＜65y | 1 | 1.38[0.63,2.92] | ND | 1 | 1.86[0.84,4.11] | ND | 4 | 0.04[-0.40,0.50] | 0.01,75 | 1 | 0.33[-0.45,1.15] | ND |
|  |  |  |  |  |  |  |  |  |  |  |  |  |
| **Treatment duration** |  |  |  |  |  |  |  |  |  |  |  |  |
| ＞12m | 3 | 1.10[0.65,1.84] | 0.52,0 | 3 | 1.60[0.92,2.76] | 0.80,0 | 3 | 0.04[-0.20,0.28] | 0.08,61 | 2 | -0.16[-0.33,0.02] | 0.18,45 |
| ≤12m | 2 | 1.57[0.08,29.55] | 0.17,46 | 2 | 0.78[0.29,2.12] | 0.83,0 | 5 | -0.05[-0.47,0.37] | 0.01,71 | 2 | 0.21[-0.49,0.91] | 0.51,0 |
|  |  |  |  |  |  |  |  |  |  |  |  |  |
| **Diabetic kidney disease** |  |  |  |  |  |  |  |  |  |  |  |  |
| CKD | 4 | 1.40[0.75,2.62] | 0.60,0 | 4 | 1.39[0.78,2.47] | 0.58,0 | 5 | 0.10[-0.195,0.392] | 0.01,69 | 2 | -0.16[-0.33,0.02] | 0.18,45 |
| DKD | 1 | 0.74[0.31,1.74] | ND | 1 | 1.27[0.53,3.04] | ND | 3 | -0.20[-0.45,0.06] | 0.31,14 | 2 | 0.21[-0.49,0.91] | 0.51,0 |
|  |  |  |  |  |  |  |  |  |  |  |  |  |
| **Impact of missing studies on overall SMD Trim and fill method** | 5 | 1.12[0.68,1.86] | 0.51 | 5 | 1.337[0.827-2.163] | 0.68 | 8 | -0.01[-0.23,0.21] | 0.51 | 4 | -0.12[-0.21,-0.02] | 0.38 |

Additional file 1: **Table S2 Effects of Self-management on CKD Risk Factors**

1. Surrogate Outcomes

|  | **Systolic Blood Pressure** | | |  | **Diastolic Blood Pressure** | | |  | **HbA1c** | | |  | **C-reactive Protein(CRP)** | | |  | **Total Cholesterol(TC)** | | |
| --- | --- | --- | --- | --- | --- | --- | --- | --- | --- | --- | --- | --- | --- | --- | --- | --- | --- | --- | --- |
|  | **No.** | **Random effects Model MD (95% CI)(mmHg)** | **Heterogeneity statistics  p-value, I^2^%** |  | **No.** | **Random effects Model MD (95% CI)(mmHg)** | **Heterogeneity statistics  p-value, I^2^%** |  | **No.** | **Random effects Model MD (95% CI)(%)** | **Heterogeneity statistics  p-value, I^2^%** |  | **No.** | **Random effects Model MD (95% CI)(mg/L)** | **Heterogeneity statistics  p-value, I^2^%** |  | **No.** | **Random effects Model SMD (95% CI)** | **Heterogeneity statistics  p-value, I^2^%** |
| **Overall summary** | 7 | -5.68 [-9.68,-1.67] | 0.01,60 |  | 7 | -2.64  [-3.78, -1.50] | <0.001,0 |  | 7 | 0.31  [-0.65,0.03] | 0.08,65 |  | 3 | -2.8  [-2.90,-2.70] | <0.01,0 |  | 5 | -0.68  [-1.39,0.04] | 0.06,88 |
| **lifestyle** | 3 | -11.23 [-19.59,-2.87] | 0.01,68 |  | 3 | -3.01  [-6.67,0.65] | 0.11,37 |  | 2 | 0.00  [-0.50,0.51] | 0.9,0 |  | 3 | -2.8  [-2.90,-2.70] | <0.01,0 |  | 3 | -1.16  [-2.97,0.65] | 0.21,93 |
| **Medical-behavior** | 2 | -3.46  [-9.02,2.11] | 0.22,32 |  | 3 | -2.07  [-5.38,1.25] | 0.22,39 |  | 2 | 0.00  [-0.12,0.13] | 0.9,0 |  | ND | ND | ND |  | 1 | -0.06  [-0.57,0.46] | 0.83,ND |
| **Multi-factorial** | 2 | -1.11  [-6.60, 4.38] | 0.70,0 |  | 3 | -2.65  [-5.48, 0.19] | 0.07,0 |  | 3 | -0.68  [-0.99,-0.36] | <0.01,0 |  | ND | ND | ND |  | 1 | -0.24  [-0.55,0.07] | 0.13,ND |

1. Health literacy

|  | **Sodium Excretion** | | |  | **Body Mass Index(BMI)** | |  |  | **Body Weight** | | |  | **Six Minute Walk Test** | | |
| --- | --- | --- | --- | --- | --- | --- | --- | --- | --- | --- | --- | --- | --- | --- | --- |
|  | **No.** | **Random effects Model MD (95% CI)(mmol/24h)** | **Heterogeneity statistics  p-value, I^2^%** |  | **No.** | **Random effects Model MD (95% CI)** | **Heterogeneity statistics  p-value, I^2^%** |  | **No.** | **Random effects Model MD (95% CI)(kg)** | **Heterogeneity statistics  p-value, I^2^%** |  | **No.** | **Random effects Model SMD (95% CI)** | **Heterogeneity statistics  p-value, I^2^%** |
| **Overa****ll summary** | 2 | -0.89[-8.23,6.46] | 0.81,0 |  | 2 | -0.25[-0.93,0.44] | 0.48,0 |  | 3 | -1.98[-4.75,0.80] | 0.16,7 |  | 3 | 0.70[0.45,0.94] | <0.01,0 |
| **lifestyle** | 1 | -5.50[-23.80,12.80] | 0.56,ND |  | 1 | -1.50[-5.04,2.04] | 0.41,ND |  | 3 | -1.98[-4.75,0.80] | 0.16,7 |  | 3 | 0.70[0.45,0.94] | <0.01,0 |
| **Medical-behavior** | 1 | -0.20[-0.90,0.50] | 0.57,ND |  | 1 | -0.20[-0.90,0.50] | 0.57,ND |  | ND | ND | ND |  | ND | ND | ND |
| **Multi-factorial** | ND | ND | ND |  | ND | ND | ND |  | ND | ND | ND |  | ND | ND | ND |

**Additional file 1: Table S3 S**tructure and Content of Self-management

| **Concept fields** | **Concept tenets** | **CALO-RE** | **CCM** | **TTM** | **CDSMP** | **SMS** | **HBM** |
| --- | --- | --- | --- | --- | --- | --- | --- |
| Attitudinal beliefs | The perceived positive benefits must outweigh the perceived negative costs of a behaviour | - | Behavioural beliefs and derived attitudes | Pro and con evaluations, decisional balance | - | - | Benefits, barriers and health motivation |
| Self-efficacy, control beliefs | Belief in one’s ability to perform a behaviour is often necessary for its execution | Concept of self-reward | Self-Management Support(emphasize the patient’s central role in managing their health) | Self-efficacy (and temptation as a negative indicator, plus self liberation) | Confidence in one’s ability to use certain skills and the belief that these skills will produce desired outcomes. | Self-management is integrated into patients’ day-to-day life | Self-efficacy (in later version |
| Normative beliefs and norm related activity influences | Belief that significant others desire one to adopt a behaviour | Plan social support/social change | Normative beliefs and motivation to comply | Helping relationship related processes | Self-efficacy affects every phase of beaviour change | - | Cues from family, friends and media |
|  | Beliefs that peers have adopted the behaviour | Prompt identification as role model/position advocate | - | Social liberation related processes | PHASE OF BEHAVIOUR CHANGE PHASE OF BEHAVIOUR CHANGE | - | - |
|  | Positive reenforcements, behavioural reminder | Identify environmental prompts which can be used to remind them to perform the behaviour | - | Reinforcement management and stimulus control processes | - | - | Cues from mass media and other sources |
| Risk related beliefs and emotional influences | One feels at risk of a defined disease/condition, with will inflict negative consequences | - | - | Dramatic relief processes | - | - | Perceived susceptibility |
| Intention setting and commitment planning | One has formed intentions and/or commitments in relation to achieving a specific behaviour | Goal setting | - | Self liberation and social liberation processes, contemplation, preparation and action stages of behavioural change | - | - | - |
